# Supplementary material for: Are carbon emissions trading and green financial instruments synergistic? -Comprehensive quantitative research based on content analysis
Source: PLoS One. 2024 Mar 7;19(3):e0298601. doi: 10.1371/journal.pone.0298601 (PMC10919601; doi:10.1371/journal.pone.0298601)
Supplement: S1 Data — (ZIP) [file pone.0298601.s001.zip › Data/地级市Z_F工作报告.zip!/╣π╢1⁄2/╔ε█┌╩╨/└┤╫╘╣1⁄2╓┌║┼í╛┬φ┐╦╩2╛▌═°í┐_2016.docx]

各位代表：

　　现在，我代表深圳市人民政府向大会作政府工作报告，请予审议，并请各位政协委员和其他列席人员提出意见。

　　一、2015年工作回顾和“十二五”发展成就

　　2015年是落实市第六次党代会部署的开局之年，是全面完成“十二五”目标任务的收官之年。我们深入贯彻落实党的十八大和十八届三中、四中、五中全会，习近平总书记系列重要讲话及对深圳工作重要批示精神，在党中央国务院、省委省政府和市委的坚强领导下，按照“五位一体”总体布局和“四个全面”战略布局，围绕建设现代化国际化创新型城市目标，坚持解放思想、真抓实干，突出质量引领、创新驱动、绿色低碳，着力稳增长、优结构、促改革、惠民生、防风险，较好地完成了市六届人大一次会议确定的目标任务。

　　初步核算，本市生产总值1.75万亿元，增长8.9%。规模以上工业增加值6785亿元，增长7.7%。固定资产投资3298亿元，增长21.4%。辖区公共财政收入7238.8亿元，地方一般公共预算收入2727.1亿元，分别增长30.4%和30.9%。进出口总额2.75万亿元，其中出口1.64万亿元，连续23年居国内城市首位。社会消费品零售总额5017.8亿元，增长2%。居民消费价格涨幅2.2%。

　　（一）着力推动经济稳增长。面对世界经济复苏乏力、外需市场低迷不振、经济下行压力贯穿全年的严峻形势，着力打好政策“组合拳”，盘活财政存量资金，出台促进经济健康发展“18条”、外贸稳增长“22条”等系列政策措施，并减免企业税负698亿元，实现经济逐季加速。新增有效投资精准发力。加快重大基础设施、重大民生工程、重点片区，以及阿里国际总部等262个重大项目建设，华星光电二期、天安云谷产业园一期等建成投产。固定资产投资增速创十七年来新高，城市更新投资增长43.1%。新兴消费需求加速释放。实施健康养老、文化体育、旅游休闲等消费培育工程，推进国家信息惠民和信息消费试点城市建设，电子商务交易额超过1.7万亿元。外贸结构持续优化。实施“一带一路”市场开拓计划，开展国际贸易“单一窗口”试点，清理规范进出口环节收费，一般贸易、服务贸易分别增长5%和15.3%。新型业态加快形成，外贸综合服务企业进出口额707亿美元，增长21%，跨境电商交易额320亿美元，增长88%。

　　（二）持续推进产业转型升级。全面优化产业结构，制定实施“互联网＋”和“中国制造2025”深圳行动计划，支持产业升级项目2350个，强化梯次型现代产业体系。继续壮大战略性新兴产业。深入推进国家战略性新兴产业发展试点，加快建设产业基地和集聚区，七大产业增加值增长16.1%，其中新一代信息技术、互联网产业分别增长19.1%和19.3%。加快培育未来产业。落实生命健康、机器人、可穿戴设备和智能装备等五大未来产业规划和政策，加快阿波罗等未来产业园发展，建设全国海洋经济科学发展示范市，成立航空航天、无人机等创新联盟。未来产业规模超4000亿元，已成为新增长点。优化发展现代服务业。制定实施金融创新“36条”、电子商务发展行动计划，新增法人金融机构20家，金融业增加值增长15.9%，占GDP比重提高0.8个百分点；旅游业总收入突破1200亿元，增长14%。现代服务业占服务业比重69.3%，提高1个百分点。大力提升优势传统产业。支持服装、家具、钟表、眼镜、黄金珠宝等产业时尚化、高端化、品牌化发展。成功举办首届深圳时装周。建成眼镜创意产业园。工业设计产值增长15%，红点产品设计奖占全国32%，中芬设计园营业收入超10亿元。

　　（三）加快国家自主创新示范区建设。出台示范区建设实施方案，全社会研发投入占GDP比重4.05%，PCT国际专利申请1.33万件，占全国46.9%，获国家科学技术奖14项，中国专利金奖获奖数占全国1/5。全省科技创新大会在我市召开。优化科技投入。实施科技创新券制度，新设创新引导基金。市区财政科技类支出209.3亿元，重点支持前沿技术、共性技术和核心技术研发，组织重大技术攻关156项。提升创新能力。新增国家、省、市级重点实验室、工程实验室、工程中心和企业技术中心等176家。组建神经科学、大数据等新型研发机构。引进“珠江人才计划”、“孔雀计划”创新团队9个和20个。强化创新创业创投创客“四创联动”。出台促进创客发展政策，建成深圳湾创业广场，举办国际创客周，打造国际创客中心。大力发展创业投资、股权众筹，新注册股权投资企业超2万家，达晨、深创投居“中国最佳创业投资机构”前两位。首只国家中小企业发展基金落户深圳。净增商事主体42.2万户，增长24.5%。

　　（四）全面深化改革开放。完成44项年度重点改革任务，36项国家试点项目取得重要进展。加快政府职能转变。推进简政放权，全面取消非行政许可事项，取消、转移、下放市级行政职权192项。公布32家市直部门、10个区权责清单。深化商事制度改革，率先实施“多证合一、一照一码”制度，开展企业名称自主申报登记等三项试点。加速前海蛇口自贸片区建设。推动法治、金融、投资贸易规则等六大领域创新，31项改革成果纳入广东自贸区60条创新经验，14项在全省复制推广。增加值突破1000亿元，税收超过170亿元，合同利用外资217亿美元。自贸新城大会战“十大战役”成效显著，完成建设投资308亿元，相当于前五年总和。积极参与“一带一路”建设。出台实施方案，支持企业参与沿线国家建设，一批项目列入国家重大项目库，完成承包工程84.9亿美元，增长94%，实际对外投资增长272%。

　　（五）着力提升城市功能。针对城市建设中的薄弱环节，加大投入力度，推进重大项目建设。加快建设交通基础设施。轨道交通6、8、10号线等7个项目动工，在建线路总长211公里，福田地下轨道交通枢纽建成使用。深圳机场实现24小时通关，新开国际航线5条。深中通道、坂银通道等开工建设。拆除二线关口，实施16个二线关口交通优化和127个拥堵道路改善工程。加快推进环境优化工程。完成26项环境基础设施提升改造。制定实施总投资816.5亿元的治水提质五年计划。新建成污水管网335公里、新开工724公里。完成深圳湾水域14个排污口整治，开工建设6.6公里滨海休闲带西段工程。与东莞市联合启动茅洲河全流域治理。完成妈湾电厂深度脱硫除尘改造。全面推广使用国Ⅴ柴油，推进港口货柜车清洁能源改造和岸电设施建设。推行生活垃圾减量分类管理。东部湾区（盐田区、大鹏新区）列入第二批国家生态文明先行示范区。加快优化发展空间。开展建设用地清退。出台严查严控违法建设决定，拆除违法建筑153.6万平米，下半年违法建筑零增长。深入推进城市更新和土地整备，城市更新供应土地2.6平方公里，整备释放土地15.3平方公里。加快实施智慧城市项目。出台宽带中国示范市实施方案，新建4G基站1.7万个，新增光纤入户67.2万户。建成全市统一电子政务公共平台。

　　（六）大力发展民生事业。九类重点民生领域市区财政支出2373.5亿元，增长63.2%，118项民生实事完成116项，其余2项正在抓紧推进，6700件民生微实事全部完成。进一步提升社会保障水平。出台创业带动就业政策，超额完成就业目标任务。最低工资、最低生活保障标准提高至2030元和800元，均居国内前列。全市实施重特大疾病补充医疗保险，启动社保同城通办试点。新开工保障性住房1.76万套、竣工2.13万套、供应2.14万套。推进市社会福利中心新址项目等19项养老工程建设。优先优质发展教育事业。新改扩建23所中小学校，新增公办学位2.67万个、幼儿园学位2.25万个。实施中小学生综合素养提升计划。重点本科上线率保持全省第一。补助民办教育、学前教育28亿元，惠及44.5万名学生。清华—伯克利深圳学院正式招生，北理莫斯科大学、中山大学深圳校区获批筹建。深圳大学、南方科技大学入选省高水平大学建设计划。新增28个职业教育实训基地。大力提高医疗卫生服务能力。实施“三名工程”，引进49个国内外高水平医学学科团队，建成3家名医诊疗中心。北京肿瘤医院深圳医院、南方医科大学深圳医院建成运营，推进57项重大医疗卫生项目建设，新增病床6400张。全面提升社康中心设备配置标准，推进三级综合医院专家进社区。积极发展文体事业。当代艺术与规划展览馆主体完工，深圳艺术学校新校区建成使用。两部作品获白玉兰戏剧艺术奖。组织开展深圳读书月、市民长跑日等群众文体活动3万场。引进CBA马可波罗篮球俱乐部、八一女排等高水平职业球队，获U15全国足球锦标赛、U16与U18青运会男篮冠军。深圳运动员夺得竞走、帆板等世界冠军。不断改善社会治理。完成龙华新区街道分设。实施居住证管理条例。“织网工程”并网运行。“一社区一法律顾问”实现全覆盖。国际公益学院落户。开展安全生产整治，关停东角头、清水河油气库。实施食品药品安全工程，加大食品监测、药品抽检力度。完成219个内涝点整治。八类严重刑事案件下降12.1%，社会治安持续向好。

　　（七）强化政府自身建设。严格践行“三严三实”，加快政府职能和作风“双转变”。自觉接受人大政协监督。定期向市人大报告工作，向市政协通报情况。认真执行人大决议决定，办理建议707件、提案325件。加快建设法治政府。提请市人大审议法规议案6件，立改废政府规章15件。完成“六五”普法任务。获中国法治政府评估第一名。努力转变工作作风。按照马上就办、真抓实干的要求，加强政府工作人员作风建设，认真落实中央“八项规定”精神，厉行节约，严控“三公”经费。进一步加强廉政建设，开展明察暗访，严格监督问责，完善绩效管理和电子监察。

　　各位代表，过去一年既是本届政府履职第一年，也是完成“十二五”规划的冲刺之年。在市委坚强领导下，经过全市人民共同努力，“十二五”目标任务圆满完成，重点领域取得突破性进展。

　　一是主动创新发展理念，经济规模和质量效益实现双提升。五年来，我们牢固树立深圳质量理念，加快转变发展方式，率先进入经济发展新常态，速度稳、结构优、动力强、效益好、消耗少成为深圳发展新特征。获2014年度珠三角“九年大跨越”考核全省第一。与2010年相比，主要指标实现“八个大幅增长”。地区生产总值由9581.5亿元增加到1.75万亿元。辖区公共财政收入由3506.8亿元增加到7238.8亿元，地方一般公共预算收入由1106.8亿元增加到2727.1亿元。全社会研发投入由333.3亿元增加到709.3亿元。国家级高新技术企业由1354家增加到5524家。战略性新兴产业规模由8750亿元增加到2.3万亿元。商事主体由78万户增加到214万户。金融业总资产由4万亿元增加到8.6万亿元。资源能源消耗实现“八个明显下降”。万元GDP能耗、水耗、建设用地、二氧化碳排放量分别下降19.5%、43%、29%、21%。氮氧化物、化学需氧量、氨氮、二氧化硫排放总量，按期完成省“十二五”减排目标要求。

　　二是坚定不移推进转型升级，经济结构战略性调整取得重大突破。在国际金融危机深度发展，我市经济增速一度下滑的情况下，我们始终保持战略定力，坚持转型升级目标不变、力度不减、步伐不停，大力推动增量优质、存量优化，五年淘汰转型低端企业超1.7万家，以转型升级的主动，实现了经济发展质量和可持续发展能力双提升。产业迈向中高端。二三产业结构由2010年的46.2∶53.7优化为2015年的41.2∶58.8。战略性新兴产业成为经济增长主导力量，增加值年均增速17.4%，占GDP比重由28.2%提高到40%。航空航天、生命健康等未来产业加快发展。先进制造业占规模以上工业增加值由70.8%提高至76.1%。金融中心地位更加巩固，金融业增加值、本外币存贷款余额等主要指标居全国大中城市第三位。优势传统产业向价值链高端攀升，行业品牌建设居全国前列，工业设计、平面设计等设计产业全国领先，获iF国际设计奖连续4年居全国首位。驰名商标159个，是“十一五”末的2.6倍。领军企业持续增加。世界五百强本土企业由2家增加到4家，中国五百强企业达30家，主营收入超百亿企业65家，其中超千亿企业8家。新增境内外上市企业83家，累计321家，本地企业中小板和创业板上市总量连续9年居大中城市首位。需求结构更加优化。与2010年相比，社会投资占总投资比重超过80%，提高15个百分点。工业产品内销比重占54.2%，提高10.4个百分点。外贸结构持续优化，一般贸易占外贸比重41.5%，提高9.9个百分点，技术贸易增长241%。新的区域增长极加快崛起。15个重点区域开发建设启动两年来，完成投资1100亿元。23个基地和集聚区成为战略性新兴产业发展的重要载体。

　　三是率先建成国家创新型城市，创新成为经济发展主引擎。五年来，我们坚持将创新作为城市发展主导战略，率先提出并积极构建综合创新生态体系，全面激发大众创业、万众创新活力，推动创新从“跟跑”向“并跑”、“领跑”转变，科技进步贡献率超过60%，三次位居福布斯中国大陆创新城市榜首。创新成果密集涌现。4G技术、基因测序、超材料、3D显示等领域创新能力跻身世界前沿。实现国家技术发明一等奖“零”的突破。获国家科学技术奖励56项，获中国专利金奖、优秀奖130项，PCT国际专利申请超5万件，分别是“十一五”的1.8倍、4.5倍和3.2倍。有效发明专利累计近8.4万件，约占全国十分之一。科研成果、科技人员入选《科学》和《自然》杂志年度全球十大科学突破、十大科学界人物。创新人才加速集聚。引进“珠江人才计划”、“孔雀计划”创新团队33个和63个，“海归”人才1.8万余人。拥有全职在深工作院士13名，政府特殊津贴专家916人。2014年陈嘉庚青年科学奖5名得主中，2人来自深圳。创新载体跨越发展。建设国家超算深圳中心、国家基因库、大亚湾中微子实验室等国家重大科技基础设施。国家、省、市级重点实验室、工程实验室、工程中心和企业技术中心等1283家，约为2010年的3倍。中科院先进院、光启等45家新型研发机构异军突起。创新体系更加开放。加快融入全球创新网络，与芬兰等9个国家签署科技合作协议，与硅谷、以色列等搭建8条“创新创业直通车”，境外投资1000万元以上研发企业新增255家。微软、英特尔、三星等58个世界500强项目落户深圳。深港创新圈建设稳步推进。创新生态不断优化。制定出台创新驱动发展“1＋10”文件等系列政策。建立云计算等45个产学研资联盟和10个专利联盟。国家技术转移南方中心落户深圳。开展科技金融试点城市建设，VC/PE机构4.6万家，注册资本超2.7万亿元。柴火空间等一批众创空间蓬勃发展。高交会、国际人才交流大会、IT领袖峰会和BT领袖峰会等国际影响力进一步扩大。

　　四是深入推进改革开放，特区体制机制优势更加凸显。五年来，我们坚持问题导向、需求导向，主动谋划、积极作为，以改革开放为特区发展提供强大动力。改革取得新突破。围绕市场化、国际化、法治化，扎实推进各项改革，主动承接国家、省242项改革任务，重点领域和关键环节改革取得重大进展。率先推进商事制度改革。深化投融资管理体制改革，社会投资项目核准事项压减90%。推进行政审批制度改革，行政许可事项从2011年的487项减少至2015年的234项。开展土地管理制度改革试点，敲响“农地入市”第一拍，存量建设用地占土地供应总量超过70%。率先开展地税征管体制改革和巨灾保险试点。公务员分类管理和聘任制、国资国企、医疗卫生、社会组织、养老服务等改革不断深化。开放再上新台阶。粤港澳大湾区纳入国家“一带一路”愿景与行动，前海蛇口自贸片区挂牌成立。全面推进深港合作，加快广深港高铁、莲塘/香园围口岸等跨境基础设施建设。深莞惠、河源、汕尾“3＋2”经济圈及深汕特别合作区建设加快推进，深河、深汕共建产业园开工建设项目640个。进出口总额从2010年的3467.5亿美元增加到4425.5亿美元，实际利用外资、对外直接投资比“十一五”分别增长42%和653%。来深投资世界五百强企业270家。扎实开展援疆援藏、对口帮扶贵州四川，完成省内扶贫开发“双到”任务。前海实现新跨越。前海22条先行先试政策基本落地。跨境人民币贷款、互联网银行、赴港发债等金融创新取得突破。前海法院、国际仲裁院成立，涉外商事调解、法律查明机制等取得重大进展。5家深港合伙联营律师事务所落户，香港税务师等八类专业人士开始在前海执业。深港青年创新创业基地建成运营，汇丰、恒生等一批重大项目签约落户。前海累计注册企业6.15万家，注册资本3.25万亿元，要素交易平台19家，持牌金融机构98家。已注册的港资企业对前海GDP贡献率超过20%。

　　五是着力建设美丽深圳，绿色低碳成为城市新特质。五年来，我们积极落实建设美丽中国的战略部署，实施了大气环境、水环境、绿化美化三大提升行动，出台了大气质量提升40条、水环境治理40条等系列措施，资源节约型和环境友好型城市建设取得新进展。节能减排成效显著。率先全面实施绿色建筑标准，绿色建筑总面积3303万平米。公共机构合同能源管理节能改造面积1136万平米。淘汰黄标车及老旧车31.2万辆。推广新能源汽车3.6万辆。率先开展碳排放权交易，2014年621家管控企业碳排放强度较2010年下降34.2%。国际低碳城获“2014可持续发展规划项目奖”。空气质量大幅提升。2015年PM2.5年均浓度降至29.8微克/立方米。灰霾天数由112天降至35天，23年来最少。空气质量居国家74个重点监测城市前列。水环境逐步改善。“十二五”政府投资243亿元，是“十一五”3倍以上，新建污水管网1402公里，新改扩建污水处理厂17座，污水日处理能力由266.5万吨提高到479.5万吨。完成238公里河道整治，部分河流水质趋于好转。绿色福利持续增加。建成2400公里绿道，总长和密度居珠三角城市首位。建成生态景观林带8.36万亩，公园总数从689个增至911个，新增公园绿地面积1614万平米。深圳湾公园、盐田海滨栈道等成为市民休闲新胜地。

　　六是加快推进特区一体化，城市现代化国际化水平迈上新台阶。五年来，我们认真落实国家新型城镇化战略，出台提升城市发展质量决定和国际化城市建设行动纲要，推动有质量的深度城市化，城市综合服务功能进一步提升。原特区内外发展协调性不断增强。实施两轮特区一体化3年计划，着力推进规划布局、基础设施和基本公共服务等“六个一体化”，过去五年原特区外投资8899亿元，占全市总投资70%以上，原特区外发展水平显著提升。城市承载力稳步提高。深圳机场第二跑道建成使用，T3航站楼建成并实现无差错转场，旅客年吞吐量3972万人次。深圳北站、东站、坪山站建成使用，厦深铁路、广深客运专线开通运营。地铁运营和在建里程389公里，公交日均客流量突破千万人次。沿江、博深等高速公路建成通车。梅观高速等6条高速路、梧桐山隧道等11个收费站取消收费，打通断头路162条。西气东输二线正式供气，岭澳核电二期、鲲鹏变电站扩建工程投入运营。实施城市更新项目330个，供应用地10.7平方公里，整备释放土地154平方公里。蝉联国内“最互联网”城市，成为国家首批“宽带中国”示范城市和信息惠民试点城市，互联网普及率、无线宽带覆盖率分别为86.2%、90.2%，主要公共场所实现WiFi上网免费。城市国际影响力进一步扩大。成功举办第26届世界大学生夏季运动会，赛会规模和项目数量为历届之最，创造了“不一样的精彩”。成立国际交流合作基金会，新增国际友城和友好交流城市38个。联合国教科文组织在我市设立高等教育创新中心。国际班轮航线255条，集装箱年吞吐量跃居全球第三。

　　七是全力实施惠民工程，民生福祉持续提升。五年来，我们加大投入力度，系统推进12项重大民生工程，全市九大类重点民生领域财政支出6734亿元，年均增长29.7%。国家确定涉及我市的65项基本公共服务全部落实。社会保障能力不断增强。社会保障和就业投入356亿元。失业保险、生育保险、地方补充医疗保险扩展到非户籍人员。新增安排保障性住房24万套，发放人才住房补贴19.7亿元。全面实施住房公积金制度。养老床位从3597张增至8359张。教育卫生事业发展提质提速。教育投入1349亿元，是“十一五”的2.4倍。率先成为省教育现代化先进市。高等教育实现跨越发展，南方科技大学、香港中文大学（深圳）建成招生，深圳吉大昆士兰大学等10所特色学院加快建设，在校全日制大学生由7万人提高到9.5万人。基础教育普惠均衡发展，新增中小学学位13.2万个、幼儿园学位14.8万个，普惠性幼儿园825所，2015年68.7%基础教育学位供给非深户学生。医疗卫生投入599亿元，是“十一五”的3倍。引进72个高水平医学学科团队，国家级、省级医学重点学科由14个增至78个；香港大学深圳医院等建成运营，三级医院从9家增至25家，三甲医院由3 家增至10家，病床数从2.3万张增至3.7万张，新增执业医生6900名。医院—社康中心双向转诊服务体系基本形成，580家社康中心开展家庭医生服务。文体旅游事业蓬勃发展。建成龙岗大运中心等26个重点文体设施。广泛开展全民健身运动，引进ATP、WTA国际网球公开赛等高水平赛事，连续举办中国杯帆船赛、深圳国际马拉松赛。12部作品获国家“五个一工程”奖，《人文颂》在海内外成功巡演。深圳荣膺“全球全民阅读典范城市”，蝉联“全国文明城市”称号。发起设立中国文化产业投资基金，国家对外文化贸易基地落户，文博会成交额实现翻番。年接待游客超1.1亿人次。社会治理水平稳步提高。发布实施城市公共安全白皮书，食品药品抽检总量五年提高一倍，110刑事治安总警情数下降47.5%。新建社区服务中心668家，实现全覆盖。获“全国和谐社区建设示范市”称号。社会组织超1万家、专业社工8230人、注册志愿者120.9万人。国防动员工作取得新成效，连续荣获“全国双拥模范城市”。创设并成功举办四届中国慈展会。此外，审计、信访、统计、民族、宗教、对台、侨务、口岸、民防、气象、档案工作取得新进展。

　　各位代表，深圳经济特区“十二五”改革发展成绩来之不易，这是党中央国务院、省委省政府和市委坚强领导的结果，是在历届市领导班子和一代又一代特区建设者打下的坚实基础上，全市人民继往开来、团结拼搏的结果。在此，我代表深圳市人民政府，向为特区建设作出卓越贡献的老领导、老同志致以崇高的敬意！向全市人民，向给予政府工作大力支持的人大代表和政协委员，向各民主党派、工商联、各人民团体和社会各界人士，向中央和省各部门、各驻深单位，向驻深解放军、武警官兵，向关心和支持深圳发展的港澳同胞、台湾同胞、海外侨胞和国际友人，表示衷心的感谢！

　　各位代表，过去五年，我们主动把握发展规律、创新发展理念、破解发展难题，坚定不移地打造“深圳质量”，在继承中创新、在创新中发展、在发展中突破，实现了总量和质量“双提升”、动力和活力“双增强”、结构和效益“双优化”，为我市未来发展打下了坚实基础。深圳经济特区的丰富实践和全市人民的伟大创造，给予我们宝贵启示：

　　——必须始终高举中国特色社会主义伟大旗帜。旗帜指引方向，道路决定命运。特区的成功探索，生动诠释了中国特色社会主义的巨大优越性，更加坚定了我们的道路自信、理论自信、制度自信。站在新的历史起点，我们要全面准确贯彻落实习近平总书记系列重要讲话精神，以中国特色社会主义政治经济学重大原则指导深圳实践，以中华民族伟大复兴的中国梦激励特区人民团结奋进，在落实“四个全面”战略布局中创造新业绩。

　　——必须始终勇担改革开放排头兵的历史使命。改革开放是决定中国命运的关键一招，是特区的根和魂。我们聚焦市场化、国际化、法治化和前海蛇口开发开放，排浪式深化改革，全方位扩大开放，为特区发展提供了强大动力。改革进入攻坚期和深水区，我们要解放思想、牢记使命，发扬敢为天下先的精神，改革不停顿、开放不止步，以更大的勇气与智慧推进治理体系和治理能力现代化，努力为全国改革开放再立新功。

　　——必须始终发挥创新驱动的引领作用。抓创新就是抓发展，谋创新就是谋未来。我们深入实施创新驱动战略，着力完善综合创新生态体系，全面激发大众创业万众创新的巨大能量，创新成为引领发展的第一动力，率先实现了发展动力转换。面对全球竞争新挑战，我们要抢抓新机遇，着力推进以科技创新为核心的全面创新，新中求进，进中突破，争创一流，加快打造全球领先的创新之城。

　　——必须始终保持追求卓越的质量自觉。速度终有上限，质量永无止境。我们按照中央把发展立足点转到质量效益上来的要求，推动“深圳速度”向“深圳质量”转变，努力以思想理念的创新、发展方式的转变和转型升级的先行赢得战略主动，实现了有质量的稳定增长、可持续的全面发展。面对经济发展新常态，我们要坚持质量发展不动摇，在中高速增长中迈向中高端水平，增创特区发展新优势。

　　——必须始终坚持可持续发展的生态文明理念。生态环境是生存之本、发展之基。我们着眼人与自然和谐共生，大力推动绿色发展、循环发展、低碳发展，用更少的资源消耗、更低的环境成本，创造了更多更好的发展成果，走出了一条经济发达地区绿色低碳发展之路。面对资源环境紧约束，我们要像保护眼睛一样保护生态，像对待生命一样对待环境，加快建设资源节约型和环境友好型社会，推动生态文明建设再上新台阶。

　　——必须始终践行以人民为中心的发展思想。民之所望，政之所向。我们坚持发展为了人民、发展依靠人民，把为民惠民作为工作的出发点和落脚点，不断增加公共产品和服务供给，努力率先全面建成小康社会。面对广大市民对美好生活的新期待，我们要始终把人民放在心中最高位置，盯住短板、精准发力，持续增进市民福祉，促进人的全面发展，加快建设幸福美好家园。

　　各位代表，我们清醒地认识到，城市快速发展中的短板和软肋不容忽视。去年底发生的光明新区“12?20”特别重大滑坡事故，造成严重的生命财产损失，我们深感痛心和自责。按照党中央国务院、省委省政府的要求，市委市政府全力以赴组织实施抢险救援，开展安抚安置和善后工作，全面排查整治安全生产隐患，积极配合国务院调查组的调查。痛定思痛，我们必须在教训中警醒、在反思中整改，针对城市公共安全的薄弱环节，举一反三，铁腕整治，坚决杜绝重特大事故发生。与此同时，我们必须高度重视发展中的其他突出问题和挑战。一是经济社会发展中的深层次问题和结构性矛盾错综复杂，改革攻坚任务十分繁重。二是土地、水等资源约束趋紧，要素成本上升较快，对特区长远发展构成严重制约。三是人力资源数量不足、结构不合理，创新型领军人才、技能型人才相对短缺，难以适应新时期发展的新需求。四是教育、医疗、住房等供给跟不上人口快速增长的需要，与市民群众的期盼相比还有较大差距。五是原特区内外发展不协调、不平衡问题依然存在，违法建筑量大面广，城市管理精细化水平不高。六是部分河流和海域水质污染还比较严重，污水管网欠账较多，重大环保设施落地难，环境治理任重道远。七是政府职能转变还不到位，行政效率有待提高。一些政府工作人员法律意识、责任意识、服务意识和担当精神不强，思想僵化、不严不实等问题比较突出。腐败案件时有发生，廉政建设和作风建设亟待加强。对此，我们将以“三严三实”的精神采取有效措施，切实加以改进。

　　二、“十三五”发展的主要思路

　　各位代表，“十三五”时期，是我国转型升级的关键时期和全面建成小康社会的决胜阶段，世界经济在深度调整中曲折复苏，新一轮科技革命和产业变革蓄势待发。综合研判国内外形势，深圳仍处于可以大有作为的重要战略机遇期，同时也存在一系列矛盾和风险。面对新形势、应对新挑战，我们必须始终把握认识新常态、适应新常态、引领新常态的大逻辑，深入推进供给侧结构性改革，打造深圳经济特区质量型增长内涵式发展的升级版。

　　我们要高举中国特色社会主义伟大旗帜，全面贯彻党的十八大和十八届三中、四中、五中全会精神，深入落实习近平总书记系列重要讲话和对深圳工作重要批示精神，根据省委省政府和市委六届二次全会部署要求，围绕“三个定位、两个率先”和现代化国际化创新型城市建设目标，按照“五位一体”总体布局和“四个全面”战略布局，牢固树立创新、协调、绿色、开放、共享发展理念，以提高发展质量和效益为中心，依托“创新驱动、质量引领、互联融合、协调均衡、绿色低碳、开放共赢、共建共享、文化强市、依法治市、市场导向”十大战略路径，重点抓好十个方面工作，加快形成引领经济发展新常态的体制机制和发展方式，使“五大发展理念”在深圳经济特区落地生根、开花结果。

　　——我们要深入实施创新驱动发展战略，加快建设国际领先的创新型城市。把创新摆在发展全局核心位置，加快全面创新改革试验，完善综合创新生态体系，形成以创新为主要引领和支撑的经济体系和发展模式，抢占未来发展制高点。增强自主创新能力，加强源头创新、开放创新、协同创新，加快从跟随式创新向引领式创新转变，提升创新发展能级。强化企业创新主体地位，实行严格的知识产权保护制度，激发全社会创新活力和创造潜能，打造国际创客中心。优化人才发展的生态环境和服务体系，构筑人才高地，建成更高水平的国家自主创新示范区，打造具有世界影响力的一流创新中心。

　　——我们要更好服务全国全省发展，加快建设更具辐射力带动力的全国经济中心城市。坚持深圳质量，继续推进经济结构战略性调整，强化企业核心竞争力，巩固提升战略性新兴产业，加快未来产业发展，推进现代服务业高端化，促进优势传统产业转型升级，构建更具竞争力的现代产业新体系，打造世界级新兴产业创新发展策源地，建设国际化金融创新中心、物流枢纽和消费中心，在更大范围、更高层级集聚配置资源，不断增强经济特区辐射带动能力。

　　——我们要努力打造国际一流信息港，加快建设信息经济为先导的智慧城市。深入落实“中国制造2025”和“互联网＋”深圳行动计划，推进信息产业化、产业智能化、城市智慧化。坚持基础设施、产业发展、应用服务“三位一体”建设，加强新一代信息技术研发和产业化，以跨界融合催生新业态，建立技术水平高、渗透力强的信息技术产业体系，构建发达的信息基础设施，广泛运用互联网、大数据、云计算等先进信息技术，重构产业链、创新链、价值链，全面拓展信息经济空间。

　　——我们要努力转变城市发展方式，加快建设协调均衡的现代化城市。尊重城市发展规律，提升城市发展质量，促进原特区内外、东西部地区协调发展。优化空间布局，实施东进战略和中轴提升战略，提升西部发展层级，优化“三轴两带多中心”布局，形成东西均衡、南北协调、周边协同的发展格局。推进特区内外一体化，加大对原特区外政策支持力度，加快基础设施建设，推进重点区域开发，打造新的区域增长极。提升综合交通枢纽功能，完善城市交通网、慢行交通系统和对外通道。提高市政设施现代化水平，构建清洁高效的现代能源体系、安全优质的供水保障体系，加快地下综合管廊建设，提高发展的系统性、均衡性、持续性。

　　——我们要率先形成绿色发展方式和生活方式，加快建设绿色低碳的生态文明城市。大力推进绿色、低碳、循环发展，建立系统完整的生态文明制度体系，争创国家生态文明试验区。发展低碳经济，创建国家低碳发展示范区。强力推进生活垃圾分类减量和无害化处理，加强再生水、各种废弃物再利用。全面促进资源集约节约利用，实施全民节能行动计划，提高能源利用效率。加大自然生态系统和环境保护治理力度，改善水生态环境，提升大气环境质量，加强土壤和噪声污染整治，积极打造“公园之城”，提升城市自然资本价值，促进人与自然的和谐共生。

　　——我们要不断提高对外开放质量和水平，加快建设更具竞争力影响力的国际化城市。积极推进高水平双向开放，促进要素有序流动、资源高效配置、市场深度融合，建设合作共赢的开放之城。主动落实“一带一路”国家战略，实施“一带一路”市场专项计划。加快与沿线国家产业融合和互补发展，深化人文交流合作。推进粤港澳大湾区建设，加强与港澳的深度合作，促进珠江口两岸协同发展，推进深莞惠及河源、汕尾“3＋2”经济圈建设，促进泛珠三角等更广范围区域合作。加快自贸片区建设和前海开发开放，建设国家金融业对外开放试验示范窗口、世界服务贸易重要基地和国际性枢纽港。扩大服务业对外开放，健全境外投资管理服务体系，优化外经贸发展环境，推进外贸发展转型升级，提升对外经济合作水平，构建对外开放新格局。

　　——我们要始终坚持人民主体地位，加快建设更高质量的民生幸福城市。持续加大民生投入，增加优质公共产品和多元化服务供给，完善公共服务体系，有序推进基本公共服务均等化，率先全面建成更高水平的小康社会。推动教育有质量全面发展，以开放合作提升高等教育，以优质特色强化基础教育，以政策扶持优化民办教育，以国际标准构建现代职业教育体系。促进医疗卫生扩量提质，增加优质医疗卫生服务供给，提升市民健康水平。加强社会保障，提高市民就业和收入水平，完善社会保险、社会福利和住房保障制度。牢固树立安全发展理念，全面实施城市公共安全白皮书，严明责任、堵塞漏洞、加强监管，不断强化公共安全保障能力，全力构筑城市安全屏障。加强社会治安综合治理，完善立体化治安防控体系，提升社会治安共管共治能力。保障食品药品安全，建设国家食品安全城市。增强应急和防灾减灾能力，健全各类突发公共事件的预防预警和应急处置体系，提高城市应急响应和恢复能力，努力实现市民安全、城市安定。

　　——我们要坚持物质文明和精神文明两手抓，加快建设具有鲜明特色的社会主义文化强市。大力培育和践行社会主义核心价值体系，加强思想道德建设，丰富城市人文精神，争创全国文明城市标兵。推进文化创新，实施“深圳文化创新发展2020”，打造文化品牌，完善公共文化服务体系，深化文化体制改革，加强文化交流合作，发展现代文化产业，不断壮大创新型、智慧型、力量型、包容型城市主流文化，推动文化大发展大繁荣，彰显特区社会主义文化影响力和软实力。

　　——我们要全面推进依法治市，加快建设一流法治城市。自觉运用法治思维和法治方式推动改革发展，率先落实依法治国各项任务，全面实现社会主义市场经济法治化，完善立法体制，加强重点领域立法。健全依法行政和决策机制，创新行政执法机制，强化对行政权力制约监督，打造法治政府。建设诚信深圳，推进全民尊法学法守法，深入开展法治创建活动，改进公共法律服务，建设法治社会，加快建设公平公正安定有序的法治环境。

　　——我们要着力推进治理体系和治理能力现代化，加快建设全面深化改革先锋城市。以经济体制改革为重点，加快完善各方面体制机制，突出供给侧结构性改革，健全使市场在资源配置中起决定性作用和更好发挥政府作用的制度体系，率先在政府职能转变、国资国企改革等重点领域和关键环节实现突破，破除一切不利于科学发展的体制机制障碍，形成一批可复制推广的改革成果，种好改革开放试验田，当好改革开放排头兵，建成更具改革开放引领作用的经济特区。

　　三、2016年主要工作

　　2016年是“十三五”开局之年，也是深圳“城市管理治理年”。今年经济社会发展的主要预期目标是：本地生产总值增长8—8.5%，居民人均收入与经济增长基本同步，城镇居民登记失业率控制在3%以内，居民消费价格涨幅3%左右。需要说明的是，8—8.5%的经济增长区间调控目标，既考虑了深圳质量型增长更具韧性潜力，发挥对全国全省经济增长支撑作用的需要，又考虑了各种风险因素积聚交织的影响和全面深化改革、转方式优结构的需要。要实现上述目标，必须认真落实市委六届二次全会部署， 按照“五破五立”要求，增强忧患意识、树立底线思维，坚定信心，扭住关键，狠抓落实。

　　（一）更加注重质量和效益，促进经济持续稳定增长。牢牢把握新常态下经济发展新趋势，以质量引领发展新常态，以质量赢得竞争新优势，适度扩大总需求，推动投资、消费、出口更加协调拉动经济增长。继续扩大有效投资。增加优质供给，加大重大基础设施、重大产业项目投资力度，加快重点区域、新兴产业基地和集聚区开发建设，规划建设国际会展中心等519个重大项目，探索政府与社会资本合作模式，全年固定资产投资增长18%左右，社会投资占比保持在80%以上。着力释放消费潜力。抢抓消费升级机遇，继续实施消费热点培育工程，发展新兴消费，加快国家电子商务示范市建设，电子商务交易额达2万亿元。支持企业参加大型展会、建立内销网络，提高国内市场占有率。推进华强北等商圈升级改造，加快建设罗湖国际消费中心，社会消费品零售总额增长9%左右。进一步提高外贸质量。实施优进优出战略，推进中国（深圳）跨境电子商务综合试验区建设，支持企业开拓境外市场，扩大自主知识产权和高附加值产品出口，加快发展外贸综合服务平台，服务贸易占对外贸易总额比重21%左右。落实进口贴息政策，鼓励进口技术含量高的成套设备，建设跨境电商直销体验中心。加快保税区、出口加工区转型发展。

　　（二）更加注重供给侧发力，推动更高层级转型升级。突出供给侧结构性改革，提高供给体系质量，提升全要素生产率，以供给创新激发新兴需求，以需求升级拉动供给提质。优化政策供给，支持企业发展。出台支持企业发展政策措施，加大正税清费力度，全面落实“营改增”政策，清理行政审批中介收费，多措并举，降低企业成本。释放一批土地空间和产业用房。用好政府投资引导基金。实施大型龙头企业培育引进计划，力争年营业收入超千亿企业达到10家左右。全面落实支持中小微企业健康发展措施。优化产业供给，提升市场竞争能力。加快发展信息、生物、航空航天、海洋经济等战略性新兴产业和未来产业，推进高端医疗装备、北斗卫星应用、石墨烯等重点领域研发和产业化。积极创建国家级制造业创新中心，加快坂雪岗科技城等3个省市共建智能制造示范基地建设。促进现代服务业跨界融合，大力发展会计、设计、咨询等专业服务业。深入推进金融改革综合创新试验区建设，强化区域性股权交易中心功能，组建金融投资控股平台，新增法人金融机构15家以上，健全金融风险防控体系。推进军民融合发展。实施新一轮企业技术改造。优化供给保障，提高产品服务质量。坚持深圳质量深圳标准，着力构建广义质量和标准体系，主动参与国家标准和国际标准制定，建设标准国际化创新型城市，推动深圳成为全球重要的标准高地和认证检测中心。以高标准打造产品服务高质量。

　　（三）大力实施创新驱动发展战略，构建创新发展新优势。推出一批政策措施。制定实施建设国际一流创新型城市的决定及配套政策，推动出台国家自主创新示范区条例。建立创客引导基金，新增50个创客空间、10个创客服务平台。建设一批创新载体。瞄准国际科技前沿，建设国际化新型研发机构。推进未来网络实验室和移动互联网安全技术等国家工程实验室建设，新增和提升创新载体100家以上。实施一批重大科技项目。围绕信息通讯、智能装备、节能环保、生物技术、新材料等重点创新领域，组织实施国家、省、市重大攻关项目290项，支持企业创新项目1000项以上。鼓励企业、高校、科研机构加强国际科技合作。培育一批高新技术企业。实施国家高新技术企业培育计划，力争国家高新技术企业累计超过6000家。引进培养一批高素质人才。推进人才工作立法，制定更具吸引力的人才政策。深入实施“孔雀计划”，引进20个以上海内外高层次创新团队、500名以上海外高层次人才。加大专业型人才和实用型人才培养引进力度，新增12万以上技能人才。办好首届中国深圳海外创新人才大赛。

　　（四）深化重点领域和关键环节改革，破除体制机制障碍。加快政府职能转变。简政放权、放管结合、优化服务，开展市场准入负面清单改革试点，全面推行部门权责清单制度。继续清理行政许可和服务事项，创新审批方式，实行跨部门协同和网上并联审批。推进“强区放权”改革，健全城市执法体制。推进经济体制改革。坚持市场导向，探索研发投入、新产品应用等政策创新。深化土地供给制度改革，完善规划用地和土地出让政策。继续深化商事制度、国资国企等改革。积极推进金融改革创新，争取“深港通”尽快实施，进一步完善多层次资本市场。深化社会事业领域改革。完善公共服务多元供给机制，统筹推进户籍、教育、养老等领域改革。积极落实国家精简“五险一金”政策。深化医药卫生体制改革，鼓励社会办医。深化文化体制改革，推动传统媒体和新兴媒体融合发展。健全生态文明制度体系。加快落实大鹏半岛生态文明体制改革总体方案。探索环境执法和监测体制改革。完善碳排放权交易制度。

　　（五）落实国家“一带一路”和自贸试验区战略，构建对外开放新格局。出台并落实服务“一带一路”倡议实施方案。推进沿线市场拓展专项计划，争取更多项目纳入国家重大项目库。积极搭建经贸合作平台，举办第九届APEC中小企业技术交流暨展览会、中国（深圳）华人华侨产业交易会等展会，推动中国（深圳）—印尼电子产业园等对外合作园区建设，办好巴布亚新几内亚深圳产品展销中心。支持企业参与沿线国家重要港口、信息基础设施等建设。加强与沿线国家在文化、旅游、生命健康等领域的交流，拓展友城网络，深化与喀什等重要节点城市合作。力争与“一带一路”沿线国家年度双边贸易额超5000亿元。加快前海蛇口自贸片区建设。探索建立更短的“负面清单”和更高标准的投资贸易规则，加快现代服务业开放创新，出台深港跨境金融创新政策。推进自贸新城大会战，推动桂湾金融先导区建设、前海保税港区扩区，建成蛇口太子湾邮轮母港等项目，完成固定资产投资360亿元。扎实推进区域合作。积极推进粤港澳大湾区建设和泛珠三角区域合作。探索深港边境地区开发合作，加快莲塘/香园围口岸建设和深圳河四期治理。深化深莞惠、河源、汕尾“3＋2”经济圈合作和对口帮扶，加快深汕特别合作区建设。继续做好援疆、援藏和帮扶毕节、甘孜工作。

　　（六）打响城市管理治理攻坚战，促进城市安全发展。立足精细化管理，着力治理“城市病”，提升城市公共安全保障能力。强化安全监管和隐患整治。理顺安全生产监管体制，建立城市公共安全技术研究院，完善公共安全形势分析制度。依法强化企业主体责任，落实政府监管责任，切实做到领导干部“一岗双责”，以最严格的标准、最严密的制度、最严厉的处罚、最严肃的问责加强安全生产管理。加大公共安全投入，新设150亿元城市公共安全专项资金，全面提升城市公共安全保障能力。彻查安全隐患，强化余泥渣土受纳场、原“二线”插花地、垃圾填埋场、危险边坡、地面坍塌、道路交通、消防火灾、危化品和易燃易爆品、特种设备等重点地域领域安全防范和专项整治。启动12个地面坍塌隐患整治项目，完成102个内涝点整治。创建国家食品安全城市，强化食品药品生产流通全过程监管。深化城市安全“科技护城墙”建设，加强反恐防暴，完善网络和信息安全保障机制，提升应急救援专业化水平。健全安全隐患和事故举报奖励机制。坚决杜绝重特大安全事故，保障人民群众生命财产安全。坚决控停违建。落实严查严控违法建设“1＋2”文件，以拆促停、疏堵结合、分类处理，确保实现“零增量”、“减存量”。整治交通拥堵。启动沙河西路快速化改造、春风隧道等工程建设，推进侨城东路北延、布罗通道等项目前期，打通科苑大道等41条断头路。启动滨海沙河西立交、滨河新秀立交、罗芳立交等交通节点改造，制定北环皇岗立交、北环彩田立交等节点改造方案。开展南山科技园、福田中心区等片区交通整治，全面完成二线关口交通综合改善工程。坚持公交优先，优化公交线路65条以上，新增公交专用道80公里，加强地铁公交接驳，开展福田罗湖慢行交通系统建设试点。大力开展“治水提质”等攻坚行动。新建成污水管网1000公里，建成福田污水处理厂，开工建设沙井污水处理厂二期，完成62公里河道整治。进一步提升市容环境。深入实施“蓝天工程”，强化挥发性有机物综合整治，提高靠港船舶低硫油使用比例，加强机动车尾气治理，年内开始永久限行黄标车，公交大巴电动化率提高到70%，PM2.5年均浓度继续保持较低水平。

　　（七）着力提高城市建设质量，增强城市综合服务能力。高标准建设交通基础设施。建成轨道交通7、9、11号线，新投入运营107公里，地铁总运营里程达到285公里，加快6、8、10号线和七段延长线建设进度，推动赣深客专开工。加快深中通道、外环高速、坪盐通道等工程建设，续建高速公路97公里。做好海滨大道一期、梅观高速市政化等项目工作。推进深圳机场扩建工程，开通5条国际航线，打造枢纽机场。统筹推进地下综合管廊规划建设。加强资源能源保障。推进清林径引水调蓄、樟坑径液化石油气等工程建设。新开工100个居民小区优质饮用水入户工程。新建改造310公里燃气管道，新增13万用户。加速打造国际一流信息港。建设国家新型智慧城市标杆市，加快信息开放共享，推进下一代互联网建设和规模商用，全面完成城域网IPv6升级改造，进一步提高光纤入户率。持续推进生态环境建设。继续做好国家低碳城市试点，强化公共机构节能工作，新增绿色建筑面积800万平米。完善垃圾回收利用体系，推动建筑工业化，提高工业、建筑等废弃物资源化水平。新建改建公园50个。做好第19届国际植物学大会筹备工作。盘活存量土地资源。完善城市更新政策，扩大整村统筹、片区统筹试点，力争全年更新供应土地2.2平方公里、整备释放土地11平方公里。强力推进闲置土地处置工作。落实征地返还用地三年实施计划。有序扩大原农村集体建设用地入市范围。

　　（八）补短板提质量，提升民生保障水平。完成12项重大民生工程投资300亿元，办好116件民生实事，继续实施“民生微实事”。加快教育事业发展。新改扩建中小学校30所，新增中小学学位3.09万个，新增普惠性幼儿园60所。实施高校卓越发展计划，全面推进深圳大学、南方科技大学高水平大学建设，加快中山大学深圳校区、天津大学—佐治亚理工深圳学院、深圳技术大学筹建，力争北理莫斯科大学获批招生。借鉴德国职业教育双元制，大力引进优质资源，推动职业教育高水平国际化发展。新增10个职业教育实训基地，减免职业院校全日制学生学费。实施劳动者素质提升计划，加强终身职业教育。支持民办教育发展。继续实施医疗卫生“三名工程”。启用萨米国际医疗中心（深圳），建成2家基因检测技术应用示范中心，加快中山大学深圳医院、平湖医院等15个重大项目建设，推进市第二儿童医院等33个项目前期工作，新增床位3000张。引进10个以上高水平医学学科团队，推进与广州中医药大学等合作办医。积极运用大数据技术，发展智慧医疗、精准医疗。完善公共卫生服务体系。推广家庭医生服务。加大社会保障力度。实施高校毕业生就业促进和创业引领计划，新增就业8万人。推进社保同城通办。研究完善住房政策体系。加快推进原“二线”插花地、棚户区改造，新开工和筹集保障性住房6万套以上、竣工5万套、供应4万套。推进市养老护理院等社会福利项目建设，加大帮扶力度，完善困境儿童基本生活费补贴制度，积极发展妇女、儿童、老龄、残疾人等社会福利事业。

　　（九）加强社会建设和文化建设，提升城市软实力。提高社会建设水平。加快建设全市统一的公共信用信息管理系统。完善“织网工程”，优化公共法律服务，促进调解、仲裁、行政裁决、行政复议等纠纷解决机制与诉讼有机衔接，加强社会矛盾纠纷风险预警和排查化解。支持工会、共青团、妇联等人民团体发挥枢纽型组织作用。促进和规范社会组织发展。完善居住证制度。落实全面放开二孩政策。开展慈善信托试点。强化基层治理。完善社区治理制度，减轻社区工作站负担。优化社区服务中心综合服务功能。建立市内经济欠发达社区帮扶机制。加快政企、社企分开，推动股份合作公司向现代企业转型。探索业主大会法人登记制度。提升居民参与社区治理和建设积极性。促进文化事业繁荣发展。争创第五届“全国文明城市”。出台基本公共文化服务指导标准，加快美术馆新馆、青少年足球训练基地、中国设计博物馆等文体设施规划建设。建立“城市文化菜单”，办好读书月、创意十二月等品牌活动。筹办“一带一路”国际音乐节、国际设计周等交流活动。构建现代文化产业体系，培育“文化＋”新型业态，加强国际数字出版基地等国家级平台建设。加快旅游产业发展，改善旅游环境，创新旅游产品。推动体育事业发展，办好ATP和WTA网球公开赛、中国杯帆船赛等国际赛事。

　　（十）从严治政，提高政府服务质量和效率。坚持依法用权、廉洁从政、高效服务，加快建设为民务实清廉政府。推进依法行政。自觉接受市人大工作监督、法律监督和市政协民主监督。坚决执行市人大及其常委会决议决定，密切与市政协沟通协商，切实做好建议提案办理工作。认真听取各民主党派、工商联、无党派人士和人民团体的意见，主动接受群众、媒体和审计监督。落实国家法治政府建设五年实施纲要，开展“七五”普法工作。创新政府服务方式，运用互联网、大数据，提供更加便捷的服务。切实改进作风和加强反腐败工作。我们必须发扬钉钉子精神，锲而不舍、驰而不息，严格践行“三严三实”要求，强化审计监督和绩效管理，以更高标准、更严举措正风肃纪。对群众反映突出的热点难点问题，做到件件有落实、事事有回音；对不作为、乱作为、推诿扯皮的政府工作人员，要严格问责。要进一步加强廉政建设，对腐败零容忍，依法依规依纪严肃查处各类违法乱纪行为。同时要积极探索建立有利于干事创业的激励机制和容错机制，努力打造一支政治坚定、敢于担当、勤政廉洁的干部队伍。

　　各位代表，新常态蕴含新机遇，新时代开启新征程。让我们更加紧密地团结在以习近平同志为总书记的党中央周围，在省委、省政府和市委的坚强领导下，高举中国特色社会主义伟大旗帜，解放思想，脚踏实地，真抓实干，勇当“四个全面”排头兵，努力建成现代化国际化创新型城市，共同创造全市人民更加幸福美好的未来，为实现“三个定位、两个率先”目标和中华民族伟大复兴中国梦作出新的更大贡献!
